# *Psidium guajava* in the Galapagos Islands: Population genetics and history of an invasive species

**DOI:** 10.1371/journal.pone.0203737

**Published:** 2019-03-13

**Authors:** Diego Urquía, Bernardo Gutierrez, Gabriela Pozo, María José Pozo, Analía Espín, María de Lourdes Torres

**Affiliations:** 1 Universidad San Francisco de Quito (USFQ), Colegio de Ciencias Biológicas y Ambientales, Laboratorio de Biotecnología Vegetal, Campus Cumbayá, Quito, Ecuador; 2 Department of Zoology, University of Oxford, South Parks Road, Oxford, United Kingdom; 3 Galapagos Science Center, Universidad San Francisco de Quito and University of North Carolina at Chapel Hill, San Cristobal, Galapagos, Ecuador; Washington University, UNITED STATES

## Abstract

The threat of invasive plant species in island populations prompts the need to better understand their population genetics and dynamics. In the Galapagos islands, this is exemplified by the introduced guava (*Psidium guajava*), considered one of the greatest threats to the local biodiversity due to its effective spread in the archipelago and its ability to outcompete endemic species. To better understand its history and genetics, we analyzed individuals from three inhabited islands in the Galapagos archipelago with 11 SSR markers. Our results reveal similar genetic diversity between islands, and the populations appear to be distinct: the islands of San Cristobal and Isabela are genetically different while the population of Santa Cruz is a mixture from both. Additional evidence for genetic bottlenecks and the inference of introduction events suggests an original introduction of the species in San Cristobal, from where it was later introduced to Isabela, and finally into Santa Cruz. Alternatively, a second introduction in Isabela might have occurred. These results are contrasted with the historical record, providing a first overview of the history of *P*. *guajava* in the Galapagos islands and its current population dynamics.

## Introduction

Invasive plant species are a threat to natural ecosystems and biological diversity [1, 2]. Studies have shown that they can reduce the local biodiversity, modify the compositions of resident communities and change nutrient cycling processes [3,4,5]. Upon invasion of a new ecosystem, invasive species have the capacity to spread, consolidate and negatively affect native flora and fauna, even leading to their extinction [1]. Islands are particularly vulnerable to invasive species because of their isolation from mainland and generally low biodiversity [6]. This susceptibility is most evident for endemic species, given their high degree of specialization to their ecological niches [6]. These species are usually less genetically diverse, have weak crossing barriers and unspecialized pollinators, all of which make them more susceptible to invaders [7].

The Galapagos Islands are an example of such an isolated ecosystem vulnerable to invasive species due to their unique biodiversity and high biological endemism [8, 9]. This archipelago is a particularly interesting scenario for the study of invasive species because of its recent and well documented history. The Galapagos were inhabited by humans during the 1830s, but human presence has been reported since their discovery in 1535 [10]. During their history, a total of 821 alien plant species have been introduced into the islands [9, 11], and some of them have been thoroughly documented due to their close connection with the human colonization process. One such species is the common guava (*Psidium guajava*), a species cultivated in South Asia, Central and South America, North America and Australia for its edible fruit [12]. It is believed that *P*. *guajava* originated in Central and South America [12] and was introduced to the Galapagos Islands in the late 19^th^ century [13, 14]. It is now recognized as one of the 37 highly invasive plants in the archipelago, having settled in non-cultivated areas since the early 1900s [6, 13]. Currently, *P*. *guajava* is widely distributed in the Galapagos, growing in both disturbed areas and natural forests in the islands that host human populations: Isabela, Santa Cruz, San Cristobal and Floreana [13, 15].

Understanding invasive processes can also benefit conservation efforts in island ecosystems. Invasive species have been formally recognized as a serious threat to the Galapagos Islands’ endemic flora and fauna, which can rapidly become extinct when alien species are introduced [10, 13]. A limited number of studies have explored the impact of invasive plants in the Galapagos (e.g. one such study reported a strong negative effect of the invasive plant *Rubus niveus* on the species richness of the *Scalesia* forests [5]), highlighting the potential of using the abundance of historial records of the islands to further explore the dynamics of invasiveness. Features such as genetic diversity and population structure are important factors when examining the origins, introduction history and invasion path of an alien species, including the number of introduction events [16].

Here we analyze the genetic diversity and population structure of *P*. *guajava* populations on the Isabela, Santa Cruz and San Cristobal islands in the Galapagos (Ecuador) to understand the history and current status of this species in the archipelago. The combination of the well documented introduction history of the common guava in the Galapagos and the relation to its genetic diversity and population structure can provide a model on how a plant species invades and spreads on an island ecosystem.

## Materials and methods

### Sampling and DNA extraction

**Fig 1:**
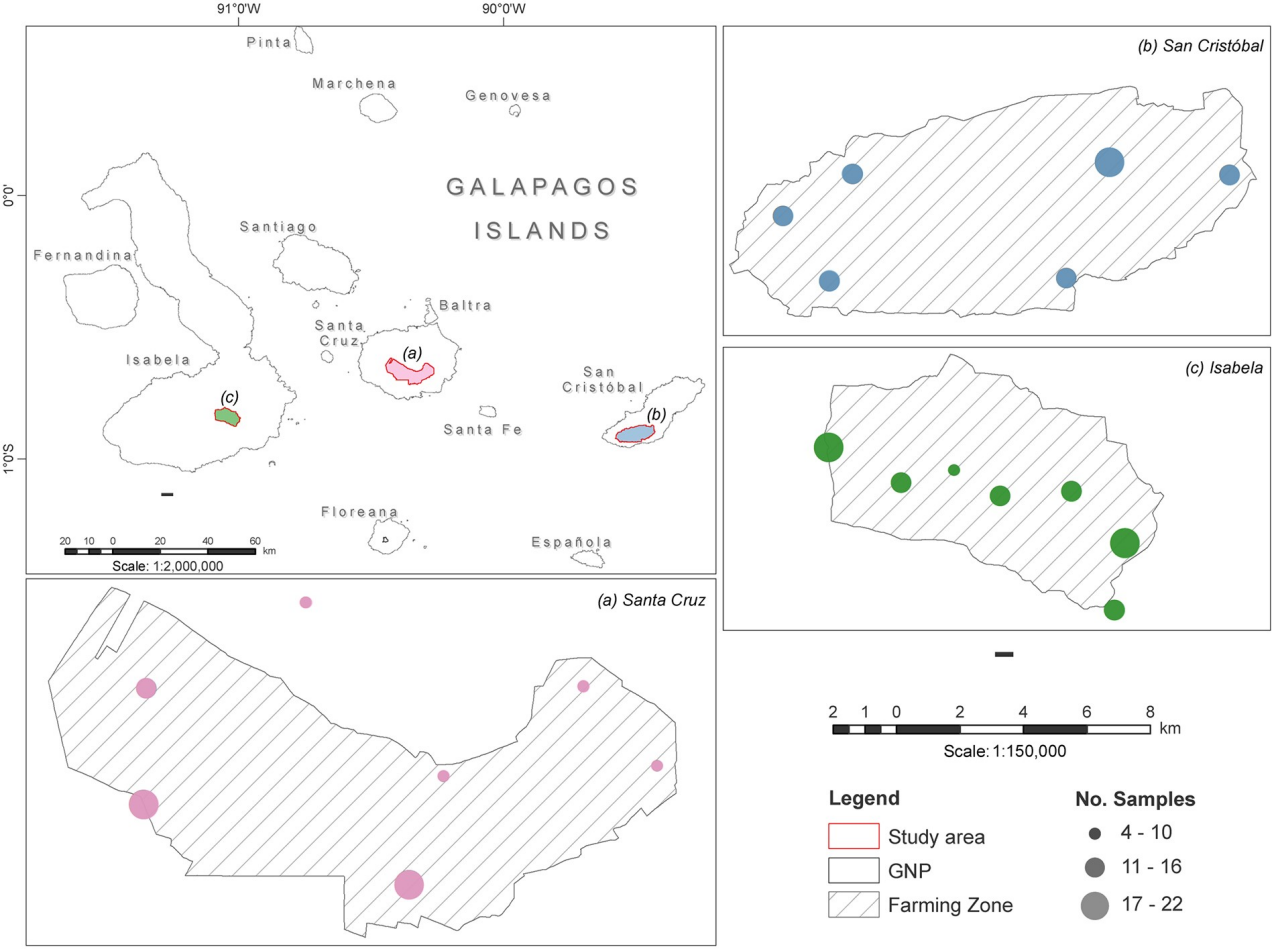
Map representing the sampling sites in three islands from the Galapagos archipelago, part of the Galapagos National Park (GNP): Isabela, Santa Cruz and San Cristobal. The diameter of each mark is proportional to the number of samples obtained from each site.

A total of 269 *P*. *guajava* individuals were sampled from selected locations of three islands in the Galapagos archipelago: 94 samples were obtained from San Cristobal (shortened SCY), 80 from Santa Cruz (shortened SCZ) and 95 from Isabela (shortened ISA). The taxonomic identity of the samples was confirmed through morphologic examination and comparison with voucher specimens from the Charles Darwin Foundation CDS Collection (Santa Cruz, Galapagos) and the Universidad San Francisco de Quito Herbarium (Cumbaya, Ecuador). Due to the historical link between *P*. *guajava* and human settlements, the sampled sites encompass each island’s farming zone (Fig 1), where populated areas and agricultural activities are widespread. Sampled individuals were separated by a minimum distance of 100m to minimize pseudosampling (given the species’ ability to propagate clonally) [12]. Two to five young leaves were collected from each individual and transported to the Molecular Biology and Microbiology Laboratory at the Galapagos Science Center in San Cristobal, where they were stored at -20°C. Collection sites were georeferenced using a Garmin E-Trex Legend HCx GPS system (Garmin International Inc., USA).

Total genomic DNA was extracted from leaves using the CTAB protocol described by Saghai-Maroof *et al*. [17]. DNA concentration and quality were assessed using a Nanodrop 1000 Spectrophotometer (Thermo Scientific, USA). DNA was then transported to the Plant Biotechnology Laboratory at Universidad San Francisco de Quito for further processing.

### Sample preparation and genotyping

Thirteen SSR markers for *P*. *guajava* developed by Risterucci *et al*. were used for this study [18]. For the PCR amplification of microsatellite loci, annealing temperatures were optimized for each set of primers; cycling conditions were 15 min at 95°C, followed by 30 to 40 cycles of 30 sec at 94°C, 90 sec at the standardized annealing temperature, 60 sec at 72°C, and a final elongation step of 5 min at 72°C. PCR products were labeled with one of four fluorescent dyes (6-FAM, VIC, PET or NED) using universal primers in a three-primer system described by Blacket, *et al*. [19]. Labeled PCR products were commercially genotyped by Macrogen (Seoul, Korea) on an ABI 3130 Genetic Analyzer (ThermoFisher Scientific, USA) using 500LIZ as a size standard. Genotyping results were analyzed using the GeneMarker software v. 2.4.0 (Softgenetics, State College, PA, USA).

### Data analysis

#### Genetic diversity and population genetics

Maps of the georeferenced sampling regions on each island were drawn using ARCGIS Desktop 10.2 (Environmental Systems Research Institute, CA, USA). For each SSR locus analyzed, the total number of alleles (A), observed heterozygosity (H_O_), expected heterozygosity (H_E_) and F-statistics were estimated on the *hierfstat* package [20] as implemented in R [21]. Private alleles (PA) were determined using the *poppr* package [22] for R. The mean allelic richness (AR), standardized to the minimum sample size (N = 80) through rarefaction, was calculated employing the *diveRsity* R package [23]. The estimates of the null allele frequencies (using the EM algorithm), for each SSR locus and population, were obtained by using the FreeNA software [24]. A Linkage Disequilibrium (LD) test and an analysis of molecular variance (AMOVA) to test the genetic differentiation between islands and between regions within each island were conducted using Arlequin 3.5 [25]. The *adegenet* R package [26] was used to evaluate the distribution of fixation indices on each island as an indicator of the proportion of inbreeding in each population. The same package was also used to perform a non-parametric Monte-Carlo test to evaluate the differences in H_E_ between islands. Finally, Hardy-Weinberg Equilibrium for each marker was tested with the *pegas* package [27] for R. Bonferroni corrections for multiple paired comparisons were applied for the LD and Monte-Carlo tests.

#### Evaluation of population structure

Pairwise F_ST_ genetic distances [28] were calculated with the *hierfstat* package; the same genetic distances, but corrected for null alleles, were obtained from FreeNA. A principal coordinate analysis (PCoA) was plotted with the *ggord* package [29] to quantify the differentiation between islands (or regions within islands) and visualize the genetic structure. Arlequin was also used to perform a Mantel test (10 000 permutations) in order to determinate whether it exists a relation between the geographic and genetic distances (Nei’s and F_ST_) among the sampled regions within all the three assessed islands. STRUCTURE 2.3.4 [30] was used to infer population structure using a Bayesian individual-based clustering approach. The program was run with an admixture model, using individual sampling islands as a prior. The potential number of genetic clusters (*K*) was evaluated between 1 and 10, with 10 independent runs performed for each *K* value. 1,000,000 Markov Chain Monte Carlo (MCMC) steps were used, with a 100,000-step burn-in period. The optimum value of *K* was evaluated using the Evanno method [31] as implemented in Structure Harvester [32], and individual membership coefficients were summarized from independent runs with the program CLUMPP [33]. The final STRUCTURE graph was plotted using the *pophelper* package [34] implemented in R. Following the same procedure, STRUCTURE plots for each island individually were also obtained (where no geographical information was included as a prior).

#### Population history of *P*. *guajava* in the Galapagos Islands

Approximate Bayesian Computation (ABC) analyses were run on DIYABC 2.0 [35] to infer the colonization patterns and introduction history of *P*. *guajava* in the Galapagos Islands. A total of 14 different colonization scenarios (S1 File) were compared through 7,000,000 simulations following a stepwise mutation model (SMM) for microsatellite loci. Scenarios 1 to 12 propose an ancestral population in one of the three islands, and two independent introduction events to the remaining two islands, with a bottleneck occurring at each introduction. Scenarios 13 and 14 propose that the population of Santa Cruz is composed of admixed origin from the remaining two islands, with the source population estimate changing between scenarios (S1 File). The simulations were created based on summary statistics that included mean number of alleles, mean genetic diversity and F_ST_ values for each population. The effective population sizes for each island (*N*_1_, *N*_2_ and *N*_3_), post-bottleneck population sizes (*N*_1b_, *N*_2b_ and *N*_3b_), times of divergence (*t*_1_ and *t*_2_) of populations and the length of bottleneck events (*d*_*b*_) (both in generations) were all drawn from uniform prior distributions (range: 10.0–10000.0). For scenarios 13 and 14, admixture rates (*r*_*a*_) were drawn from a uniform distribution (range: 0.001–0.999). Posterior probabilities of each scenario were computed using the logistic regression approach in DIYABC 2.0. The introduction times (in generations) for each island (t1 and t2, with the island identity varying between scenarios) according to the best supported scenarios were also estimated. To better understand these patterns and confirm possible introduction events, the potential of population bottlenecks in every island was inferred through the BOTTLENECK software, following both, the SMM and TPM models [36]. Heterozygosity excess or deficiencies were tested through a Wilcoxon Sign-Rank test.

## Results

### Genetic diversity of *P*. *guajava* in the Galapagos Islands

Genetic information for *P*. *guajava* individuals from three islands in the archipelago was successfully obtained for 11 of the 13 nuclear SSR markers tested (S1 Table). Two of the original 13 markers were monomorphic and therefore excluded from our analyses. The number of total alleles ranges between 40 alleles for the Isabela population to 25 alleles for the San Cristobal population. However, when counting the frequent alleles exclusively (defined here as alleles which occur at a frequency >0.05; see [37]), we found that the numbers were very similar (20–22 frequent alleles) among the three islands (Table 1). The number of exclusive alleles for each island differs greatly, ranging from 2 in San Cristobal to 15 in Isabela. The latter is also the only population that displays exclusive alleles that occur at frequencies higher than 0.05 (Table 1). The mean allelic richness was also the highest in Isabela, followed by Santa Cruz, and the lowest in San Cristobal (Table 1).

**Table 1:** Genetic diversity information of the analyzed *Psidium guajava* populations from Isabela, Santa Cruz and San Cristobal islands: Number of individuals genotyped from each island (N), number of alleles found (A), number of private alleles (PA), mean allelic richness (AR), observed heterozygosity (H_O_), expected heterozygosity/gene diversity (H_E_) and inbreeding coefficient (F_IS_). Overall results along the three islands are also shown.

Island	N	A*	PA*	AR^a^^s^	H_O_ ^a^	H_E_ ^a^	F_IS_
Isabela	95	40 (22)	15 (4)	3.586	0.106	0.284	0.621
Santa Cruz	80	35 (21)	8 (0)	3.182	0.169	0.365	0.539
San Cristobal	94	25 (20)	2 (0)	2.267	0.213	0.326	0.341
**Overall**	269	52 (21)	-	4.727	0.163	0.356	0.505

* Values between brackets are the number of alleles or private alleles with a frequency >0.05 within the corresponding island population.

^a^indicates average across the 11 SSRs analyzed.

^s^standardized for N = 80

The expected heterozygosity (H_E_) estimates show a greater genetic diversity in Santa Cruz (H_E_ = 0.365; SD = 0.202) and San Cristobal (H_E_ = 0.326, SD = 0.230), with the lowest value found in Isabela (H_E_ = 0.284, SD = 0.133) (Table 1). These differences in H_E_ between islands were not significant (given a Bonferroni correction for the test) between Isabela and San Cristobal (*p* = 0.037), but were significant between Santa Cruz and both Isabela (*p* = 0.001) and San Cristobal (*p* = 0.005). Linkage disequilibrium between markers was not found for Santa Cruz (and was spuriously found in one pair of markers in San Cristobal) but appears to be relatively common in Isabela (S2 Table). This location-dependent linkage may suggest the effect of some evolutionary forces in the Isabela population rather than linkage due to genomic proximity between markers, therefore permitting the assumption of independent segregation of markers for all posterior analyses. Hardy-Weinberg Equilibrium was also tested for all markers, indicating significant disequilibrium for most markers in all islands (S3 Table). Null alleles had a low to mid frequency along every SSR loci and population, ranging from ~0 to a maximum frequency of 0.255; along all the loci and the three populations, a null allele frequency of <0.1 appeared in the 33% of cases (i.e. locus-population pair in S4 Table), while a <0.2 frequency appeared in an 82% of cases. Null alleles were the less frequent in the San Cristobal population; meanwhile, Isabela and Santa Cruz shared similar null allele frequencies. In average, the analyzed SSR loci had a null allele frequency ranging from 0.010 (mPgCIR22) to 0.199 (mPgCIR17) (S4 Table).

The degree of inbreeding in each of the three islands, explored through the F_IS_ statistic, revealed values ranging from 0.341 to 0.621 (Table 1). The overall inbreeding coefficient for all three islands appears higher than the values for each individual island, and a visual inspection of the distribution of inbreeding coefficients shows a skewed, non-normal distribution (S1 Fig), suggesting that inbreeding could occur in a proportion of the sampled individuals.

### Population structure

The genetic differentiation of *P*. *guajava* populations across the archipelago is relatively low. Only 13% of the variation was explained by the separation between islands. When analyzing the two most divergent populations, Isabela and San Cristobal, the molecular variance explained by populations only rose to 20%, suggesting that a large degree of genetic similarities is shared between islands (Table 2). The analysis of differentiation between sampling locations within each island reveals even lower proportions of the molecular variance embedded in this level, suggesting no differentiation within each island (S5 Table). However, it should be noted that some genetic similarities within islands appear to follow an isolation-by-distance pattern, as the pairwise F_ST_ genetic distances between regions within each island or region reached considerably high values (S6–S8 Tables).

**Table 2:** Results of the analysis of molecular variance (AMOVA) performed for the *Psidium guajava* populations of Isabela, Santa Cruz and San Cristobal islands, and over the Isabela and San Cristobal populations, excluding Santa Cruz. Missing data was ignored for the AMOVA calculations.

	Three islands		Isabela & San Cristobal	
Source of variation	% of variation	*p*-value	% of variation	*p*-value
Between Islands	13.17	0.001	20.05	0.001
Between samples within Island	44.24	0.001	39.24	0.001
Within samples	42.58	0.001	40.71	0.001

**Fig 2:**
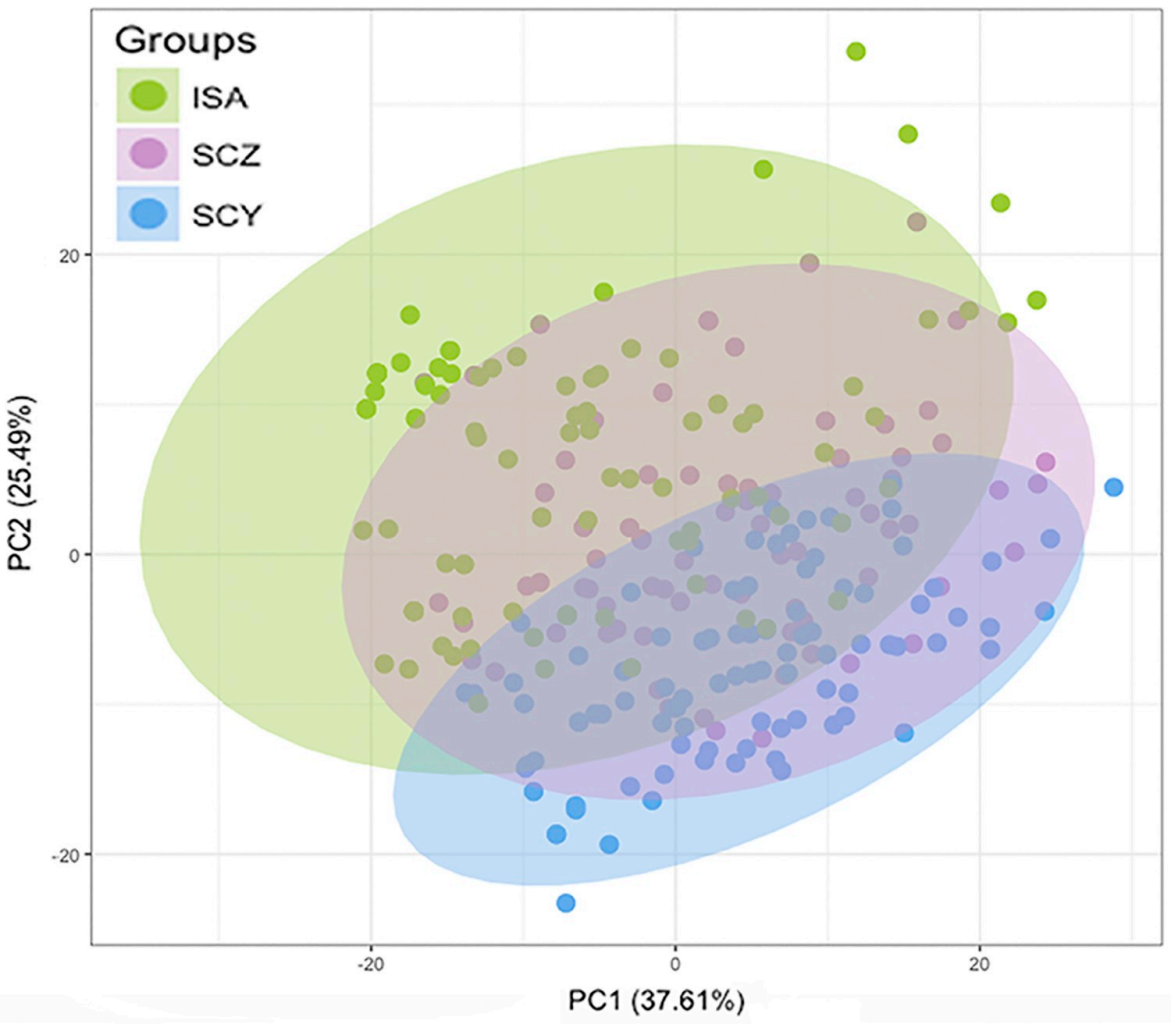
PCoA based on the genetic distances (Euclidian) found between the individuals sampled in the three islands: Isabela (ISA—green), San Cristobal (SCY- blue) and Santa Cruz (SCZ—purple).

**Fig 3:**
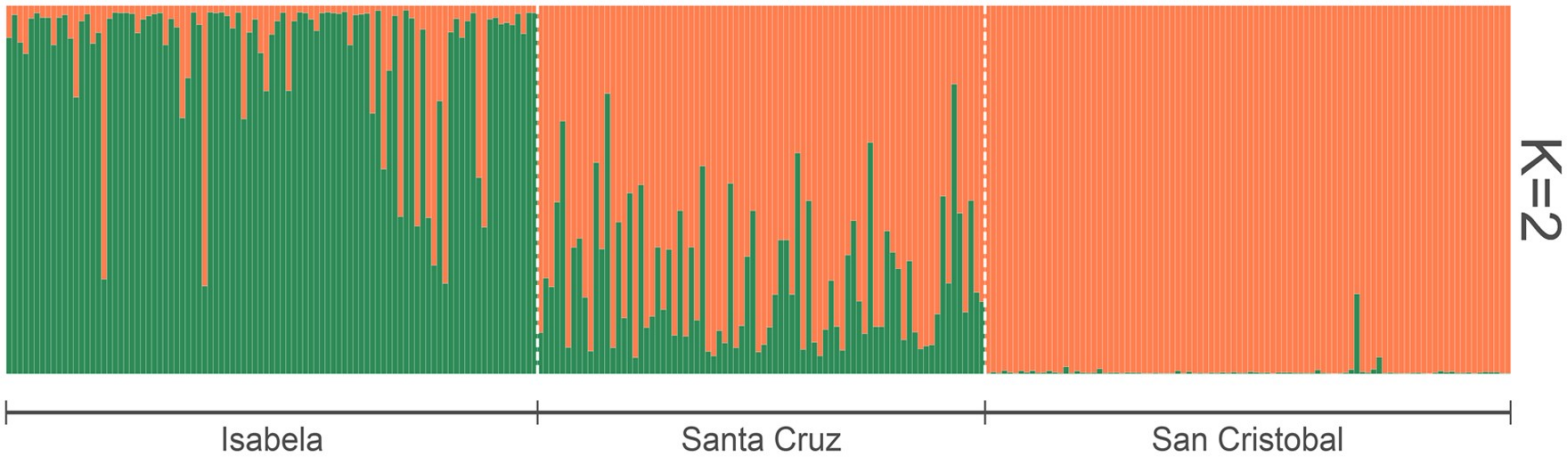
Results of the Bayesian analysis of population structure (Software STRUCTURE) under the Admixture model. The results are indicated for K = 2, this being the optimum K value (ΔK = 249). The values of K correspond to the number of clusters (represented by different colors) in which are grouped the *Psidium guajava* individuals sampled in Isabela, Santa Cruz and San Cristobal islands.

To further explore the degree of genetic structure in these populations, different approaches were considered. A PCoA based on genetic distances shows minor differentiation between islands, even when the top two principal components explain over 60% of the total variance (Fig 2). This approach shows a greater differentiation between the populations from Isabela and San Cristobal, the two furthest apart islands in our study. Furthermore, a Bayesian estimation of the number of clusters (*K*) that best explain the population structure in our data set suggested an optimal number of two lineages which cluster San Cristobal and Isabela under different groups (Fig 3, orange and green respectively). The population of Santa Cruz is shown as a combination of both gene pools, with a clear dominance of the San Cristobal lineage (Fig 3). A similar analysis reveals no defined structure within each island’s *P*. *guajava* population (S2–S4 Figs). The quantification of the differences between islands through F_ST_ genetic distances indicates that the population in Isabela is the most divergent, showing greater differences to San Cristobal (F_ST_ = 0.207) than to Santa Cruz (F_ST_ = 0.120). This pattern coincides with the geographic distances between the three islands. The genetic distance between Santa Cruz and San Cristobal is considerably lower (F_ST_ = 0.074), suggesting the more widespread gene flow between these two islands throughout the history of *P*. *guajava* in the archipelago. When corrected for null alleles, the F_ST_ distances did not change significantly. The Santa Cruz-San Cristobal F_ST_ distance remained almost the same (F_ST_ = 0.076); while, the F_ST_ values between Isabela and the other two populations, experienced a slight decrease (Isabela-San Cristobal F_ST_ = 0.183; Isabela-Santa Cruz F_ST_ = 0.096).

The geographic distances between sampling regions of the three islands were consistently correlated to Nei’s (Mantel test, *p* < 0.001, r^2^ = 0.343) and to F_ST_ genetic distances, with a lower linearity observed for the latter (Mantel test, *p* < 0.001, r^2^ = 0.152). This suggests that some isolation by distance among different populations of *P*. *guajava* found on different islands is observed.

### Population history of *P*. *guajava* inferred from genetic marker data

As an invasive species, *P*. *guajava* has been subject to one or more introduction events in the archipelago, which represent distinct demographic processes that can leave particular genetic footprints in the species’ genome [38]. We tested the possibility of a genetic bottleneck in each island under two distinct SRR mutation models and found significant evidence for a potential population expansion in Isabela (under the SMM model) and a population reduction in San Cristobal (under the TPM model) (Table 3). While these results provide some evidence for specific bottlenecks in specific islands, it also suggests that, under the lack of bottleneck events, there may have been some continuous gene flow between islands (or the archipelago and some continental source population) which reduces the signal of bottleneck events, particularly in Santa Cruz.

**Table 3:** Wilcoxon test results for the support of genetic bottlenecks in the past of the *Psidium guajava* populations of Isabela (ISA), Santa Cruz (SCZ) and San Cristobal (SCY) islands (software BOTTLENECK). Both, the SMM and TPM mutation models implemented in BOTTLENECK were used. One-tailed tests were performed in order to determinate whether the genetic bottleneck occurred because of a heterozygosity (*H*) deficiency (which may happen after a dramatic expansion on the population size) or a *H* excess (which may happen after a dramatic reduction on the population size).

	SMM Model			TPM Model		
	ISA	SCZ	SCY	ISA	SCZ	SCY
*H* deficiency (Bottleneck due population size expansion)	0.011*	0.138	0.902	0.062	0.539	0.998
*H* excess (Bottleneck due population size reduction)	0.992	0.884	0.125	0.949	0.500	0.004*
*H* deficiency or excess (Bottleneck broadly)	0.021*	0.275	0.250	0.123	1.000	0.008*

* indicates *p*-values < 0.05 which support the evidence of a genetic bottleneck.

**Fig 4:**
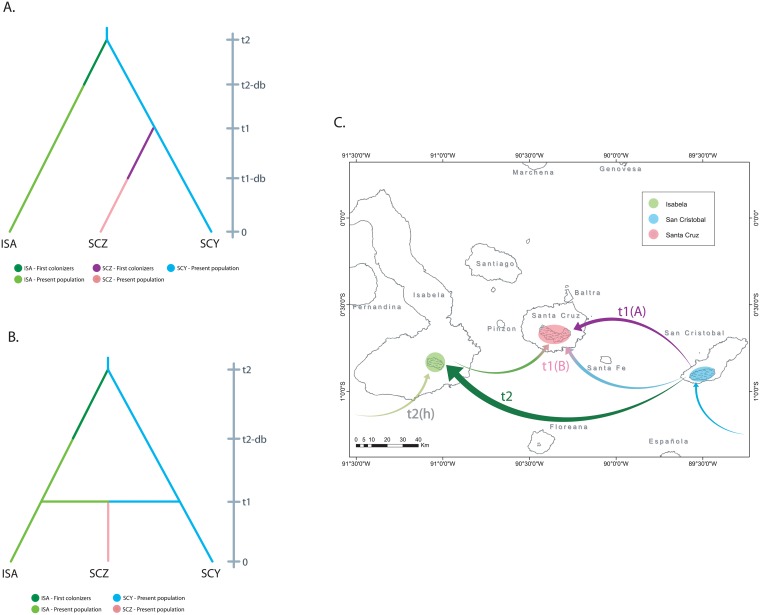
The history of the introduction of *Psidium guajava* in the Galapagos islands. The best models for the introduction history of *P*. *guajava* were estimated through Approximate Bayesian Computing (ABC) and suggest either a model of independent introductions into Isabela and later Santa Cruz from San Cristobal **(A)**, or an initial introduction into Isabela from San Cristobal followed by introduction events from both islands into Sant Cruz **(B)**. The map **(C)** shows a first introduction into San Cristobal (cyan) from an unknown source (presumably continental South America), which seeded a consecutive introduction into Isabela (t2). The population of Santa Cruz might have been formed by a single introduction from Isabela (t1(A)) or introductions from both San Cristobal and Isabela (t1(B)). It could be proposed that an independent introduction into Isabela also occurred (t2(h)) either before or after the population of San Cristobal.

The population history of *P*. *guajava* was further investigated through model testing through an Approximate Bayesian Computation (ABC) approach [35] in order to clarify the number of introduction events, the order of these occurrences and the directionality of each event. A total of 14 different scenarios were tested (S1 File), where the two best supported scenarios (posterior probabilities of 0.331 and 0.364) proposed an original introduction event into San Cristobal (Fig 4). Sub sequentially, the species might have been taken into Isabela first, and later to Santa Cruz (Fig 4A). Alternatively, the Santa Cruz population might have been formed by introduction events from both San Cristobal and Isabela (Fig 4B). According to the DIYABC estimates of the best supported scenario (scenario 11), the divergence of the Isabela population from the San Cristobal source (event t2) could date from 18 to 529 *P*. *guajava* generations ago (mean = 124 generations), depending on the model. Meanwhile, the split of the Santa Cruz population (event t1) could date from 5 to 113 generations ago (mean = 35.3 generations) (S2 File). Given the flowering time of this species at approximately two years of age [39], our estimates for divergence times appear to be inflated (since 1890, 63 generations could have occurred). These discrepancies can potentially be a product of the standing genetic diversity during each introduction event or due to multiple introductions, but given the nature of the models and data, the values should not be interpreted as formal time estimates, but rather as indicators of the relative times between the introduction events for each island. It should be noted that we only analyzed individuals from the archipelago and not from any external populations and were therefore unable to determine the source for the current populations in all three islands. It is also still unclear whether an independent introduction event might have occurred for Isabela (event *t*_2_(h), Fig 4C), associated with the high number of exclusive alleles in this population (this event could have occurred from the same continental source population or a different one). This hypothetical scenario could date the separation between the Isabela and San Cristobal populations to before the first introduction of *P*. *guajava* in the archipelago.

## Discussion

### Genetic diversity and population structure of *Psidium guajava* in the Galapagos Islands

The overall genetic diversity of *P*. *guajava* in all three islands (H_E_ = 0.356) is low when compared to the species diversity within its native range, an expected pattern for an insular invasive species [40]. According to research conducted in Brazil [41] and Venezuela [42] with *P*. *guajava* accessions from germplasm collections, the genetic diversity found in those countries (H_E_ = 0.678 and H_E_ = 0.740 respectively) is considerably higher than that found in the Galapagos *P*. *guajava* populations. This low genetic diversity in island populations is related to a founder effect, where the first plants that arrive on an island are low in number and are sampled at random from the source population. Because of this, much of the ancestral population’s diversity is left behind, lowering the possibility of obtaining heterozygote individuals [36,43,44]. It is also important to consider the size of the colonizing population, as small numbers of invasive individuals will be a smaller representation of the original population’s total genetic diversity [43].

Other plants that are known invaders in island ecosystems, such as *Cortaderia jubata* and *C*. *selloana* in New Zealand [45] and *Miconia calvescens* in several Pacific islands [46] exhibit a very low genetic diversity, as expected (H_E_ = 0.061, H_E_ = 0.095 and H_E_ = 0.110 respectively). However, other invasive species in island settings, like *Senesio madagascarensis* [47] and *Paraserianthes lophantha* [48] in Hawaii, exhibit much higher genetic diversity levels (H_E_ = 0.790 and H_E_ = 0.600 respectively), argued to be a product of gene flow or multiple introductions. By contrast, the *P*. *guajava* populations in Galapagos show an expected heterozygosity which is intermediate to these scenarios. This suggests that factors such as multiple introductions, the magnitude of the founder effect, the reproductive system of the species and the geography of the invaded ecosystem [43,49,50] could have affected the genetic diversity of the invasive *P*. *guajava* populations.

Furthermore, the *P*. *guajava* populations in different islands from the Galapagos archipelago share some features that provide an insight into the forces that have shaped their current status. The equivalent allelic diversity in all three islands, particularly if rare alleles are excluded, suggests that similar introduction dynamics have shaped these populations, or that a certain degree of homogenization through gene flow between islands has occurred. Upon closer inspection, the significantly higher expected heterozygosity index in Santa Cruz (0.365), when compared to Isabela (0.284) and San Cristobal (0.326), opens the possibility of multiple introductory events that lead to a higher genetic diversity [51]. The low heterozygosity in Isabela and San Cristobal could be explained by a low genetic variation among the first *P*. *guajava* introduced to these islands, a common occurrence during the colonization of new island ecosystems [40], and a lower number of introduction events.

The degree of differentiation among different islands is noteworthy, as it suggests that gene flow is considerably higher than might be expected for plant populations in different islands. Only 13% of the total differences occur between islands (Table 2), suggesting that gene flow between islands has occurred whether by natural or anthropogenic causes. The major differences of population structure are most likely derived from different introduction events (see below), as the recent history of the species in the archipelago provides limited opportunities for adaptive processes to produce any notable population stratification [52].

### Factors behind the success of the *Psidium guajava* invasion in the Galapagos

Despite its low genetic diversity, *P*. *guajava* has proven to be an aggressive invader in the Galapagos [15]. One way to explain this paradox addresses the reproductive biology of *P*. *guajava*. A proportion of the analyzed individuals showed higher values of the F inbreeding index (S1 Fig), and the mean inbreeding coefficients are high for the three populations (Table 1). These results may be explained, at least partially, by selfing and vegetative reproduction, which occur frequently in *P*. *guajava* [53]. Clonal propagation usually entails the formation of root suckers [54] which lead to the development of monospecific forests where individual trees show high survival rates, larger foliar surfaces and faster growth and fruiting rates [55,56]. In the initial stages of its introduction into each island, these reproductive strategies could have allowed this plant to spread rapidly [57]. Then, outcrossing, which occurs with a frequency of 35–40% in *P*. *guajava* [12], would allow the already widespread population to maintain its genetic diversity and therefore its adaptability and survivability [58,59]. Thus, this capability of combining selfing with outcrossing could be one of the key features that explain why *P*. *guajava* became a successful, widely distributed and difficult to control species in the local ecosystems. Related species such as *P*. *cattelianum* have been shown to combine clonal and sexual propagation during invasion events, as evidenced in the Hawaii archipelago [56].

Furthermore, the spread of *P*. *guajava* could have been aided by ecological factors and the disturbance of native ecosystems. In the Galapagos, abandoned farms became ideal places for this plant to grow and, due to a lack of control, to establish monotypic forests seen today on the islands [60]. Anthropogenic activities and the presence of domestic animals such as cattle, pigs and goats remove native vegetation and alter soil, creating space for the invasive species to spread [2,43,61]. These zones display a higher pH and lower amounts of carbon and nitrogen when compared to other soil types in the archipelago [62]. These conditions could provide an adequate environment for *P*. *guajava*, which is well adapted to multiple types of soil and luminosity, and can also tolerate drought conditions for up to five months [53,63]. The general climate conditions of the higher, humid zones of the islands where guavas are distributed mimic the natural environments of the species’ natural continental distribution [53], further hinting that these environmental conditions may play a key role on the success of *P*. *guajava*. Other important factors could include epigenetics [64] and phenotypic plasticity [46,65].

The success of the species in the Galapagos and its ability to propagate could be the direct cause for the lack of population structure within each island. Low levels of genetic differentiation among the regions within a single island are put into evidence by low F_ST_ values between regions (S6–S8 Tables), low percentages of genetic differentiation between regions (Table 2) and the widespread genetic admixture within each island (S2–S4 Figs). A possible explanation for these observations is a high degree of gene flow within islands, associated to the dispersion of seeds by birds such as finches, tortoises and certain lizards [66,67], as well as humans and domestic animals [68]. The small size and high number of seeds in each fruit also help *P*. *guajava* disperse with high efficiency [69]. Beyond seed dispersal, little is known about the role of potential native and non-native pollinators in the archipelago, but they could also influence the widespread gene flow inferred from our data. These factors contribute to the homogenization of allelic frequencies between regions within each island.

Due to *P*. *guajava’s* success as an invasive plant, it could pose a threat to closely related species such as the endemic congeneric guayabillo (*Psidium galapageium)* [70]. In the Galapagos, both species partially share their spatial distribution; potentially making them direct competitors and candidates for interspecific hybridization [70]. Extinction of insular plant species via hybridization between congeners has been well-established: when it occurs, it can reduce a native species’ population growth by altering its interactions with other species and affecting its reproductive and competitive success, eventually leading to its extinction [71,72]. This is exemplified by the ongoing hybridization in Socorro Island (Mexico) between native species *Psidium socorrense* and its congener *P*. sp. aff. *Sartorianum* which is resulting in the local extinction of the native species at the southern slope of the island [72]. Future studies should focus on the possible hybridization events between the *P*. *guajava* and *P*. *galapageium*.

### *P*. *guajava* in the Galapagos: The history of an invasion

The relatively recent colonization of the Galapagos Islands allows us to link the population genetics of *P*. *guajava* with the well documented historic events that describe the first human settlements on the archipelago. Most of our historical sources are based predominantly on the memories and journals kept by the first settlers and resident sailors of the Galapagos, as well as their descendants’. The information from these sources matches the historical documents obtained from the National Archives, archaeological evidence (e.g. abandoned haciendas and factories from the XIX century in Galapagos) and publications from academic historians [73,74].

Palynological research confirmed that *P*. *guajava* was present in the highlands of San Cristobal at least since the 1930’s [75]. However, *P*. *guajava* was documented in historical sources several decades prior, where early Galapagos settlers wrote about their concern of the invasive species and saw it as a potential plague [51,73,76,77]. One of the earliest mentions of *P*. *guajava* dates from the late XIX century (ca 1889–1890), where it is said that the very first three guava trees on the archipelago were planted by Manuel J. Cobos in his personal garden on San Cristobal Island for ornamental purposes. Cobos was an eminent settler of the Galapagos and was well-known throughout the archipelago. In 1870 he established a massive and profitable sugar cane plantation over the highlands of San Cristobal [73,74]. Unfortunately for the heirs of Cobos, by the 1920’s, the plantation began to be displaced by *P*. *guajava* monotypic forests [51,73]. In the 1930’s, the plague that had already become a problem in San Cristobal appeared in Floreana island, the first island colonized by humans [51,77]. Having witnessed the negative effects of this plague in San Cristobal and Floreana, the few permanent settlers of Santa Cruz tried to eradicate any plant or seedling that appeared to be *P*. *guajava*, to no success. By the 1950’s the plague had spread to Santa Cruz [51,73]. *P*. *guajava* is poorly documented in the Isabela records, but according to Lundh (2006) [51] the plague arrived at an unspecified time and persists to this day.

#### San Cristobal

The results obtained in this study provide evidence that coincides with the historical sources which suggest that the San Cristobal *P*. *guajava* population is the origin of the plague that spread over the four inhabited islands of the Galapagos. All but two rare alleles from the San Cristobal population are also present in the Isabela and Santa Cruz populations (Table 1). Furthermore, the STRUCTURE assignment coefficient plot shows that the San Cristobal population lineage (Fig 3: shown in orange) is present in over 50% of the Santa Cruz population, and to a lesser extent in the Isabela population as well. The PCoA shows the San Cristobal cluster overlapping markedly the Santa Cruz cluster and also some Isabela individuals (Fig 2). Moreover, this hypothesis is supported by the two best supported scenarios in the ABC analysis. Both of these scenarios show the San Cristobal *P*. *guajava* population as the ancestral population from which the Isabela and Santa Cruz populations derived (Fig 4).

The San Cristobal population shows evidence of having gone through a genetic bottleneck which created a transient excess of heterozygosity (Table 3), an event that occurs when the population size is reduced to a few individuals [78,79]. However, it should be considered that, despite Lundh’s [73] affirmation that the San Cristobal plague began with three guava trees planted in Cobos’ garden, San Cristobal received more settlers after Cobos from other islands within the archipelago and the Ecuadorian mainland as well. Therefore, it is possible that some of these new settlers brought new *P*. *guajava* seeds or seedlings which also contribute to the present-day San Cristobal population. This phenomenon would have increased the genetic diversity of the San Cristobal population to a certain extent but would have not obscured the previously described bottleneck due to a founder effect. The F_IS_ value estimated for this population is markedly lower than the values found for the Santa Cruz and Isabela populations. This lower level of inbreeding might be expected when severe bottlenecks occur, as is the case of the invasive plant *Miconia calvescens*, where very few individuals (or possibly a single individual) planted in Tahiti Island, started an aggressive invasion that spread over several islands of the Pacific [46].

#### Isabela

Despite the fact that *P*. *guajava* occupies a larger area on Isabela than on any other island within the Galapagos [80], there is practically no information about the history of this invasive species on the island. Lundh [51] simply mentions that the *P*. *guajava* invasion persists in Isabela and implies that the arrival of several new settlers after the 1950’s triggered the invasion on this island. Nevertheless, both historical events and our results suggest that *P*. *guajava* could have been present in Isabela several decades before the 1950’s. This island was the third to be permanently colonized by humans, following Floreana and San Cristobal [73]. Antonio Gil was one of the first settlers of Isabela, he arrived in 1897 after having lived in Floreana for four years [74]. Floreana and San Cristobal were the only islands of the Galapagos with permanent and well-established settlements and there was trade between the two islands in the late 19th century [73]. Therefore, it is possible that *P*. *guajava* was introduced from San Cristobal to Floreana during this period. From here, we could speculate that Antonio Gil, who worked with cattle, might have carried *P*. *guajava* seeds or fruit to Isabela. This statement is supported by both of the scenarios obtained from our ABC analysis which show that the Isabela population derived from the original population of San Cristobal, before the Santa Cruz population appeared (Fig 4). The AMOVA results ratify these scenarios and indicate 20% of variation between the populations of San Cristobal and Isabela (Table 2) which suggests important genetic similarities among these populations despite being located on opposite sides of the archipelago.

Both historical and genetic data point to the San Cristobal population as the predecessor of the Isabela population. However, 8 frequent alleles were found in Isabela but not in San Cristobal. Furthermore, Isabela had a higher mean allelic richness than San Cristobal (Table 1), and several Isabela *P*. *guajav*a individuals were positioned far away from the San Cristobal cluster in the PCoA (Fig 2). Also, in the STRUCTURE plot, the Isabela population is mostly clustered in a lineage that is practically absent in San Cristobal (Fig 3: shown in green). These results suggest that a second independent introduction of *P*. *guajava* to Isabela from somewhere other than San Cristobal or Floreana is possible, having an important contribution to the present-day Isabela *P*.*guajava* population and its genetic diversity. This second independent introduction could have occurred either from the same gene pool than the one that seeded San Cristobal or an independent source. In the first case, the ABC divergence time estimates for the San Cristobal and Isabela populations suggests that both islands share a common source population at some date that predates the introduction of *P*. *guajava* to the Galapagos, presumably a common source continental population. Furthermore, the new contribution of alleles from a second introduction could also have inflated the heterozygosity expected at mutation-drift equilibrium, which is very sensible to a change in the number of alleles [36], thus explaining the heterozygosity deficiency found in the BOTTLENECK analysis.

#### Santa Cruz

Santa Cruz was the last island of the archipelago to be permanently colonized, and therefore, the last island where *P*. *guajava* arrived. Settlements began in the 1910’s by former workers of the Cobos plantation in San Cristobal including a foreman, and a single settler from the Ecuadorian mainland. More people arrived in the 1920’s and 1930’s; some of them from the mainland who brought cattle with them, and some of them were workers and sailors from San Cristobal [73,74]. *P*. *guajava* could have been introduced to Santa Cruz in these decades by the abovementioned settlers but, according to historical data, the actual invasion of *P*. *guajava* in Santa Cruz didn’t begin until the 1950’s [51]. Historical data are in accordance with our best supported scenarios from the ABC analysis, which corroborate that the Santa Cruz population is the last one to derive from the ancestral population from San Cristobal (Fig 4).

Our results show that the genetic pool of the Santa Cruz *P*. *guajava* population has an important contribution not only from the San Cristobal population but also from the lineage that was independently introduced in Isabela. This can be observed in the PCoA where the Santa Cruz cluster overlaps with some individuals from Isabela (Fig 2). The STRUCTURE plot also shows a contribution of the Isabela lineage in the Santa Cruz population (Fig 3). Finally, one of the best supported scenarios in the ABC analysis (scenario B) confirms that Isabela, along with San Cristobal, contributed to the present-day Santa Cruz population. The multiple origins of the Santa Cruz *P*. *guajava* population may explain the absence of evidence for a bottleneck in this island (Table 3), since the effects of genetic bottlenecks are softened when multiple introductions occur [45,46]. The admixture of the San Cristobal and Isabela lineages in Santa Cruz, may also help explain the higher genetic diversity and number of lineages (*K* = 4, S3 Fig) found.

#### Post-invasion events

The time when the Isabela lineage arrived in Santa Cruz is unclear. During the first half of the XX century, Isabela remained isolated due the presence of a penal colony and the lack of a freighter ship serving this island regularly [73]. This fact may explain the observation of private alleles and the conservation of a separate *P*. *guajava* lineage in Isabela, as unique alleles may have been produced in a short timespan through the naturally high mutation rates of microsatellite regions and an increase in the effective population size of Isabela [81]. However, this does not explain how and when this lineage was established in Santa Cruz. Our results suggest that there was some gene flow between the populations of Santa Cruz and Isabela (F_ST_ = 0.120; 0.096 when corrected for null alleles). During the 1960’s, the penal colony was removed and more people (not only from the mainland but also from Isabela and San Cristobal) began to move to Santa Cruz, where tourism was being developed [73,76,82]. Therefore, it is possible that the arrival of *P*. *guajava* in Santa Cruz occurred at this time. The important gene flow detected between the populations of San Cristobal and Santa Cruz (F_ST_ = 0.074; 0.076 when corrected for null alleles) may be dated around this time as well. Meanwhile, the Isabela lineage is almost absent in San Cristobal (Fig 3), a fact that coincides with the lower gene flow detected between these two islands (F_ST_ = 0.207; 0.183 when corrected for null alleles). It is interesting to note that the population from Santa Cruz, which is located in the center of the archipelago, has gene flow with both the San Cristobal and the Isabela populations. On the other hand, these two populations, located on opposite extremes of the archipelago, show very little gene flow between them. Thus, gene flow between *P*. *guajava* populations from different islands seems to be correlated with their geographic locations, potentially reflecting the heightened human mobilization between islands that are closer together. It must be noted that all of the gene flow between *P*. *guajava* populations located on different islands would be presumably human-mediated, since this invasive species’ natural dispersers cannot travel across the ocean [66,67,68]. Nowadays, this type of gene flow would be completely interrupted due to the strict biosafety regulations in the Galapagos [83] which ban the mobilization of *P*. *guajava* seedlings, fruit or seeds between islands and between the mainland and the islands (Mónica Ramos, ABG, personal communication).

## Conclusions

Genetic information has increasingly become a powerful tool for the reconstruction of a population’s history and the characterization of its diversity and the forces that shape it [84]. Our current results reveal some of these processes in three *P*. *guajava* populations from the Galapagos Islands, and provide a valuable insight into the history of the invasive process of this species. However, the addition of new information to the presented framework could aid in better understanding the full extent of the risks that *P*. *guajava* represents to the local ecosystems.

Our reconstruction of the history of the invasion is backed by historical records but was insufficient to explore the source population for the Galapagos *P*. *guajava* populations. Historical sources pinpoint different provinces in mainland Ecuador as the possible source [52,74,75,82], a fact that could be further corroborated with more widespread sampling of these locations. It is also interesting to consider that Floreana Island might have played an important role in the propagation of *P*. *guajava* in the archipelago, and it remains the only inhabited island with unsampled guava populations. Further studies will address some of these questions, to continue to elucidate the history of the *P*. *guajava* invasion.

The purpose of describing an invasive process such as this is to ultimately recognize the extent of the risk it poses to the local flora and fauna, and to assist in the development of effective control and mitigation strategies. The case of *P*. *guajava* is particularly noteworthy due to the occurrence of a native member of the same genus in the archipelago: *Psidium galapageium*. The tools that have been used to explore the invasion of *P*. *guajava* could also be used to explore the effects of the co-occurrence of both species in the same spatial context. Firstly, a deeper exploration of the adaptive processes occurring on both species and their signature at the molecular level could allow us to quantify the degree to which both species compete directly with each other, and to account for the toll that *P*. *guajava* places on its endemic counterpart. Secondly, a complete analysis of the potential for hybridization between these two species can account for the potential noxious effects that *P*. *guajava* could place on the genetic diversity of the *P*. *galapageium* populations in the archipelago.

## Supporting information

S1 File Diagrams of the 14 scenarios tested through ABC analysis to infer the history of *Psidium guajava* colonization in the Galapagos Islands. Time is not shown to scale and is measured as number of generations, considering t2> = t1. Pop1/N1: Present Isabela *P*. *guajava* population. Pop2/N2: Present Santa Cruz *P*. *guajava* population. Pop3/N3: Present San Cristobal *P*. *guajava* population. N1b: Isabela first *P*. *guajava* colonizers.N2b: Santa Cruz first *P*. *guajava* colonizers.N3b: San Cristobal first *P*. *guajava* colonizers.
(PDF)

S2 File Posterior probability distributions for all parameters inferred from 70 000 simulations under Scenario 11. (PDF)

S1 Fig Histogram showing the frequency of the F (inbreeding coefficient) values observed within the 269 *Psidium guajava* individuals sampled. F values were obtained by computing the likelihood function.
(TIF)

S2 Fig Results of the Bayesian analysis of population structure (Software STRUCTURE) under the Admixture model, considering only the *Psidium guajava* individuals sampled in Isabela Island. The results are indicated for K = 2, this being the optimum K value (ΔK = 19.62). The values of K correspond to the number of clusters (represented by different colors) in which are grouped the sampled individuals. White dotted lines separate different regions.
(TIF)

S3 Fig Results of the Bayesian analysis of population structure (Software STRUCTURE) under the Admixture model, considering only the Psidium guajava individuals sampled in Santa Cruz Island. The results are indicated for K = 4, this being the optimum K value (ΔK = 39.57). The values of K correspond to the number of clusters (represented by different colors) in which are grouped the sampled individuals. White dotted lines separate different regions (GR = Granillo Rojo).
(TIF)

S4 Fig Results of the Bayesian analysis of population structure (Software STRUCTURE) under the Admixture model, considering only the *Psidium guajava* individuals sampled in San Cristobal Island. The results are indicated for K = 2, this being the optimum K value (ΔK = 1.83). The values of K correspond to the number of clusters (represented by different colors) in which are grouped the sampled individuals. White dotted lines separate different regions.
(TIF)

S1 Table Percentage of missing data for the three islands, missing data per primer and the total missing data for all individuals (269). (DOCX)

S2 Table Populations in which a significant linkage disequilibrium (LD) between pairs of loci was found, after Bonferroni Correction. The names of the linked loci appear in rows and columns, whereas population names appear as entries in the table. ISA = Isabela population; SCY = San Cristobal population. No LD was found in Santa Cruz.
(DOCX)

S3 Table Results of the Hardy-Weinberg Equilibrium (HWE) test for each loci within the three island populations (Isabela, Santa Cruz, San Cristobal). Results shown correspond to those after Bonferroni correction.
(DOCX)

S4 Table Estimate of null allele frequencies for each analyzed Psidium guajava population (Isabela, Santa Cruz and San Cristobal) and SSR locus. The mean frequencies over the three populations are shown as well.
(DOCX)

S5 Table Results of the analysis of molecular variance (AMOVA) performed within the Psidium guajava populations from Isabela, Santa Cruz and San Cristobal islands, individually. Missing data was ignored for this analysis.
(DOCX)

S6 Table Pairwise FST values between all the Isabela Island regions in which Psidium guajava individuals were sampled. (DOCX)

S7 Table Pairwise FST values between all the Santa Cruz Island regions in which Psidium guajava individuals were sampled. (DOCX)

S8 Table Pairwise FST values between all the San Cristobal Island regions in which Psidium guajava individuals were sampled. (DOCX)
