# Supplementary material for: Psidium guajava in the Galapagos Islands: Population genetics and history of an invasive species
Source: PLoS One. 2019 Mar 13;14(3):e0203737. doi: 10.1371/journal.pone.0203737 (PMC6415804; doi:10.1371/journal.pone.0203737)
Supplement: S1 File — Time is not shown to scale and is measured as number of generations, considering t2> = t1. Pop1/N1: Present Isabela P. guajava population. Pop2/N2: Present Santa Cruz P. guajava population. Pop3/N3: Present San Cristobal P. guajava population. N1b: Isabela first P. guajava colonizers.N2b: Santa Cruz first P. guajava colonizers.N3b: San Cristobal first P. guajava colonizers. (PDF) [file pone.0203737.s001.pdf]

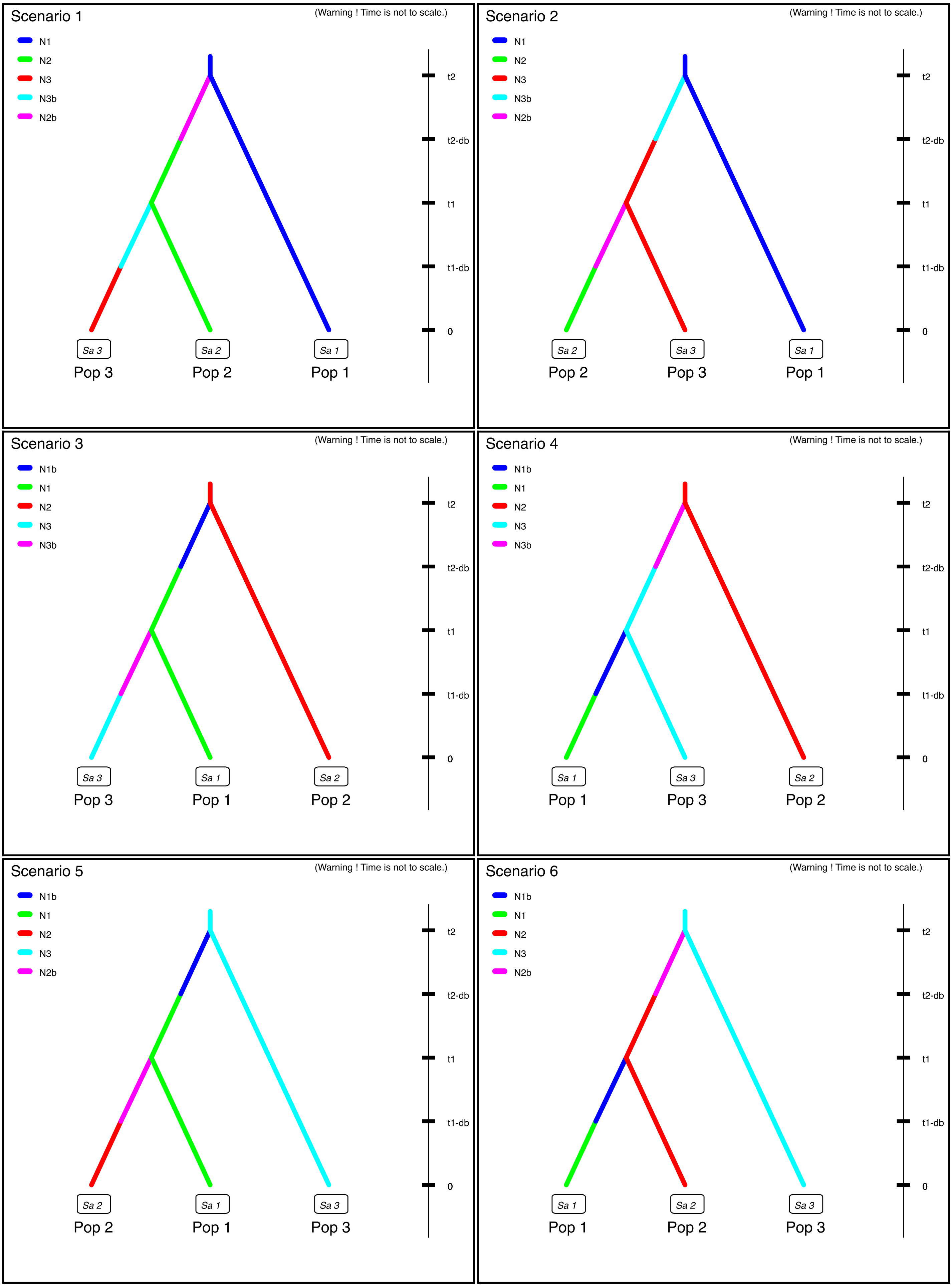
Scenario 1
(Warning ! Time is not to scale.)
N1
N2
N3
N3b
N2b
t2
t2-db
t1
t1-db
0
Sa 3
Pop 3
Sa 2
Pop 2
Sa 1
Pop 1
Scenario 2
(Warning ! Time is not to scale.)
N1
N2
N3
N3b
N2b
t2
t2-db
t1
t1-db
0
Sa 2
Pop 2
Sa 3
Pop 3
Sa 1
Pop 1
Scenario 3
(Warning ! Time is not to scale.)
N1b
N1
N2
N3
N3b
t2
t2-db
t1
t1-db
0
Sa 3
Pop 3
Sa 1
Pop 1
Sa 2
Pop 2
Scenario 4
(Warning ! Time is not to scale.)
N1b
N1
N2
N3
N3b
t2
t2-db
t1
t1-db
0
Sa 1
Pop 1
Sa 3
Pop 3
Sa 2
Pop 2
Scenario 5
(Warning ! Time is not to scale.)
N1b
N1
N2
N3
N2b
t2
t2-db
t1
t1-db
0
Sa 2
Pop 2
Sa 1
Pop 1
Sa 3
Pop 3
Scenario 6
(Warning ! Time is not to scale.)
N1b
N1
N2
N3
N2b
t2
t2-db
t1
t1-db
0
Sa 1
Pop 1
Sa 2
Pop 2
Sa 3
Pop 3

Scenario 7

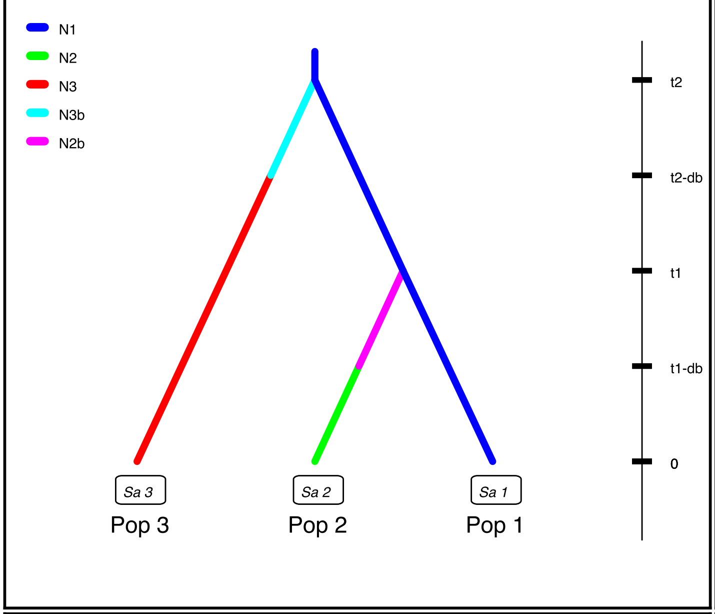

Scenario 8

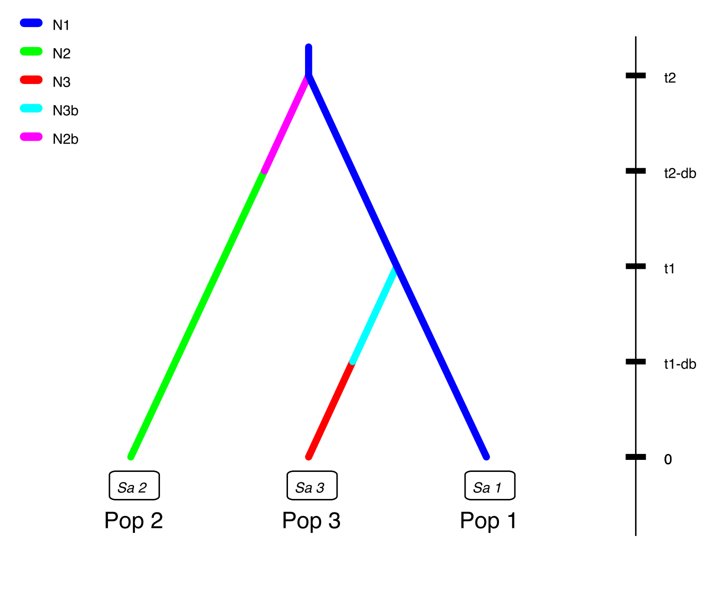

Scenario 9

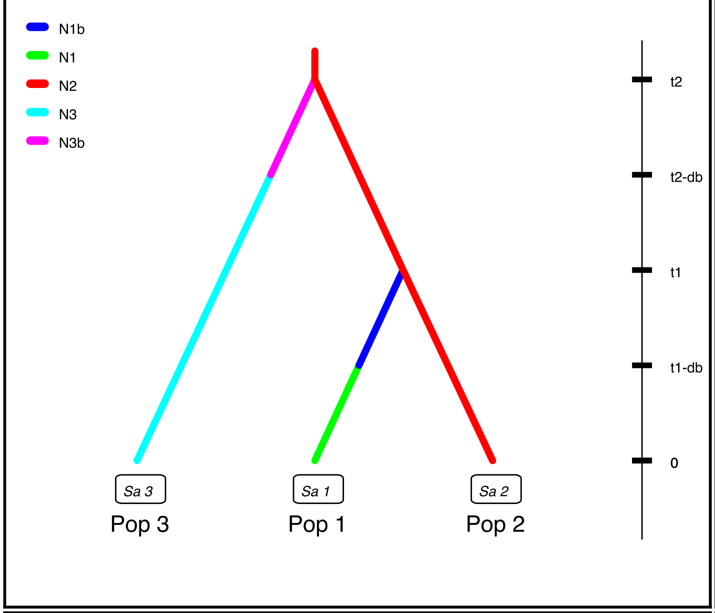

Scenario 10

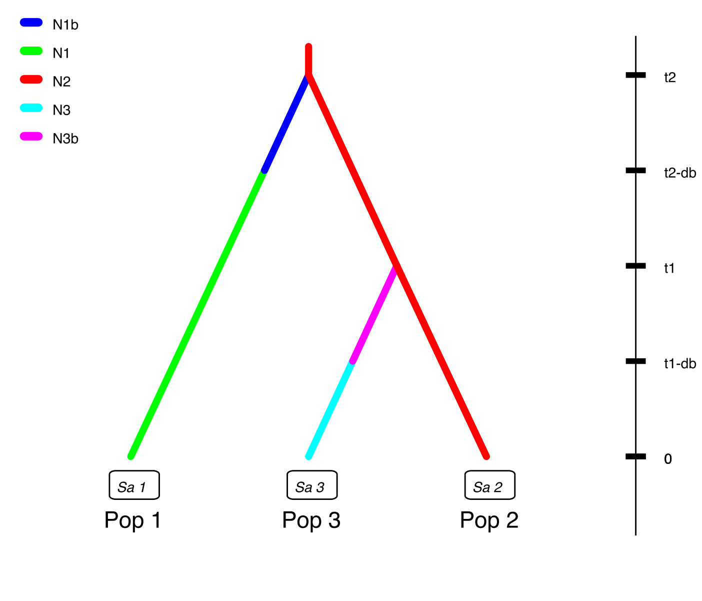

Scenario 11

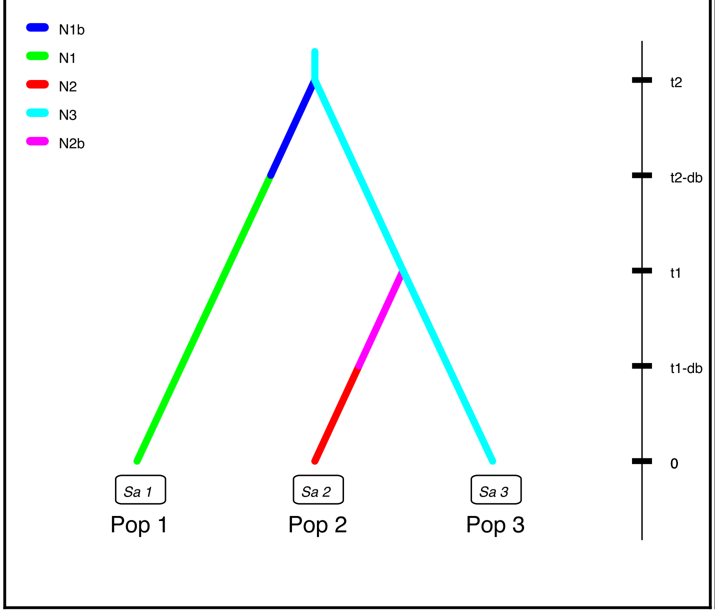

Scenario 12

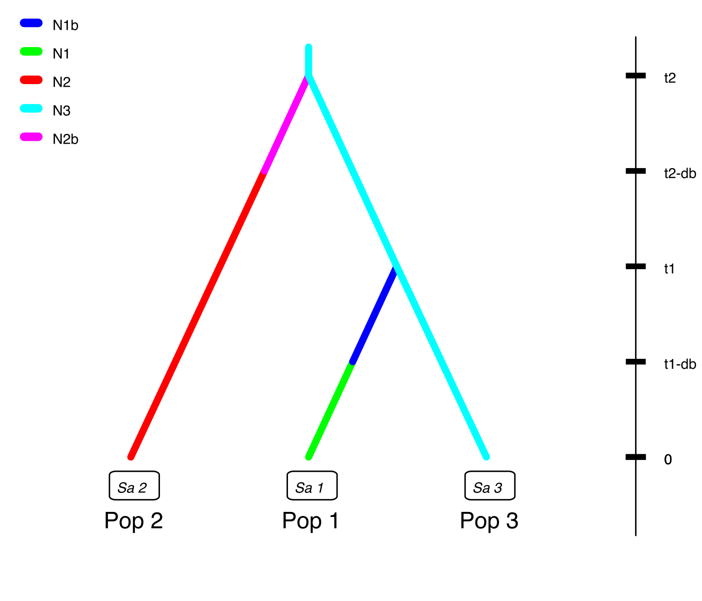

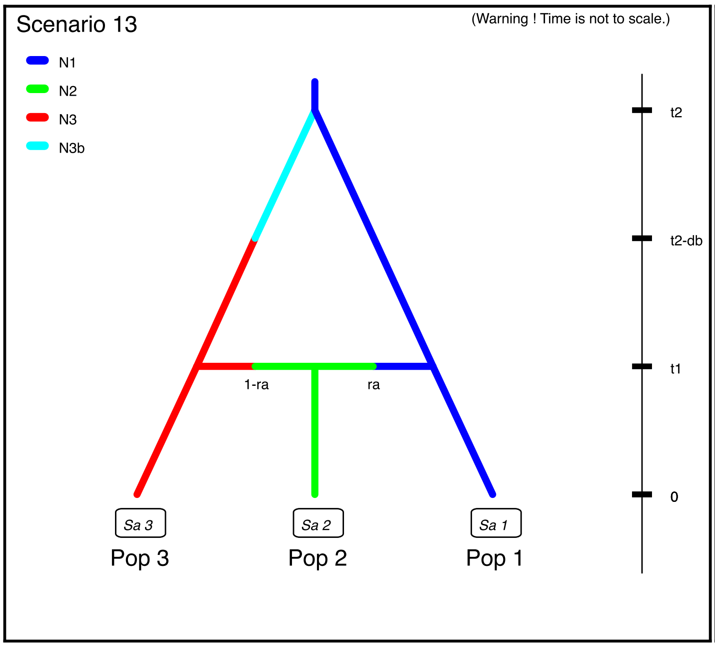
Scenario 13
(Warning ! Time is not to scale.)
N1
N2
N3
N3b
t2
t2-db
t1
0
1-ra
ra
Sa 3
Sa 2
Sa 1
Pop 3
Pop 2
Pop 1

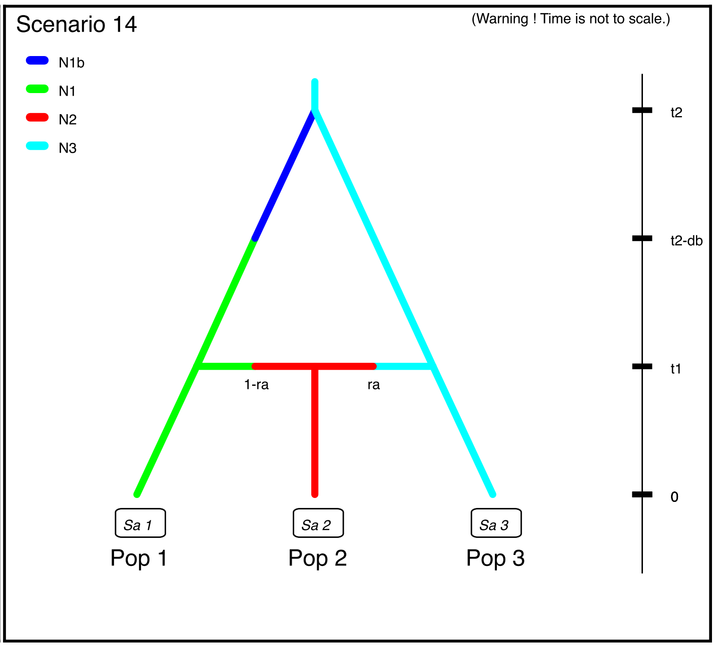
Scenario 14
(Warning ! Time is not to scale.)
N1b
N1
N2
N3
t2
t2-db
t1
0
1-ra
ra
Sa 1
Sa 2
Sa 3
Pop 1
Pop 2
Pop 3
