# Supplementary material for: Psidium guajava in the Galapagos Islands: Population genetics and history of an invasive species
Source: PLoS One. 2019 Mar 13;14(3):e0203737. doi: 10.1371/journal.pone.0203737 (PMC6415804; doi:10.1371/journal.pone.0203737)
Supplement: S2 File — (PDF) [file pone.0203737.s002.pdf]

DIYABC 2.0 output – approximate posterior probability distributions
Scenario 11

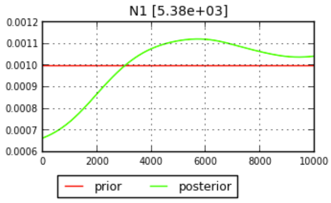

Average = 5.32e03
Median = 5.38e03
Q2.5 = 4.50e02
Q97.5 = 9.80e03

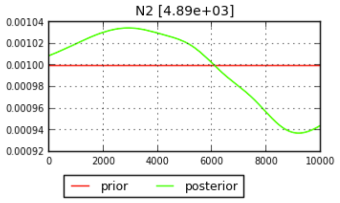

Average = 4.92e03
Median = 4.89e03
Q2.5 = 2.64e02
Q97.5 = 9.76e03

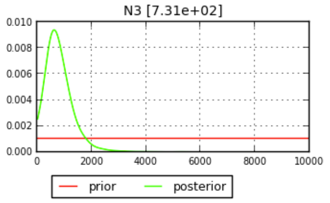

Average = 8.42e02
Median = 7.31e02
Q2.5 = 2.13e02
Q97.5 = 2.10e03

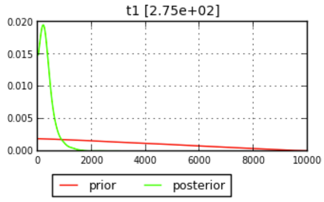

Average = 3.53e02
Median = 2.75e02
Q2.5 = 4.88e01
Q97.5 = 1.13e03

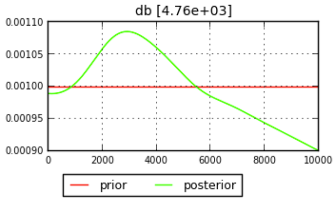

Average = 4.88e03
Median = 4.76e03
Q2.5 = 2.42e02
Q97.5 = 9.72e03

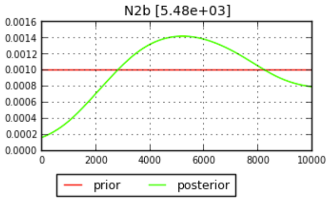

Average = 5.52e03
Median = 5.48e03
Q2.5 = 1.35e03
Q97.5 = 9.68e03

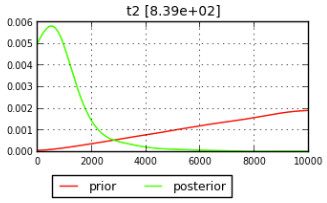

Average = 1.24e03
Median = 8.39e02
Q2.5 = 1.80e02
Q97.5 = 5.29e03

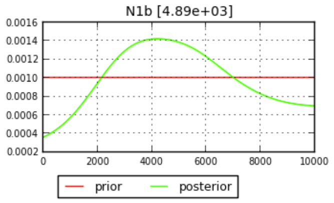

Average = 5.06e03
Median = 4.89e03
Q2.5 = 8.84e02
Q97.5 = 9.63e03

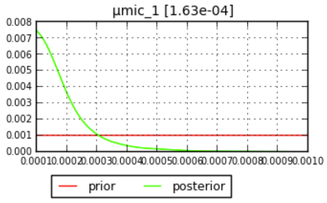

Average = 1.96e04
Median = 1.63e04
Q2.5 = 1.04e04
Q97.5 = 4.98e04

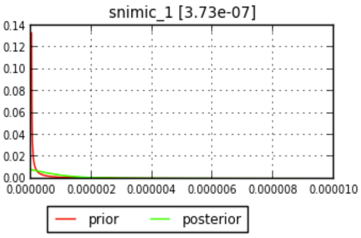

Average = 1.33e06
Median = 3.73e07
Q2.5 = 1.30e08
Q97.5 = 7.52e06
