# Supplementary figures and images for: Psidium guajava in the Galapagos Islands: Population genetics and history of an invasive species
Source: PLoS One. 2019 Mar 13;14(3):e0203737. doi: 10.1371/journal.pone.0203737 (PMC6415804; doi:10.1371/journal.pone.0203737)

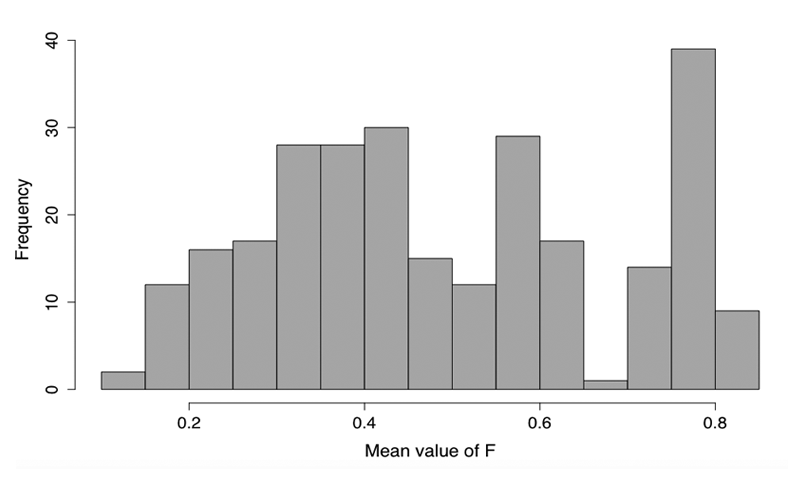

Supplement: S1 Fig — F values were obtained by computing the likelihood function. (TIF) [file pone.0203737.s003.tif]

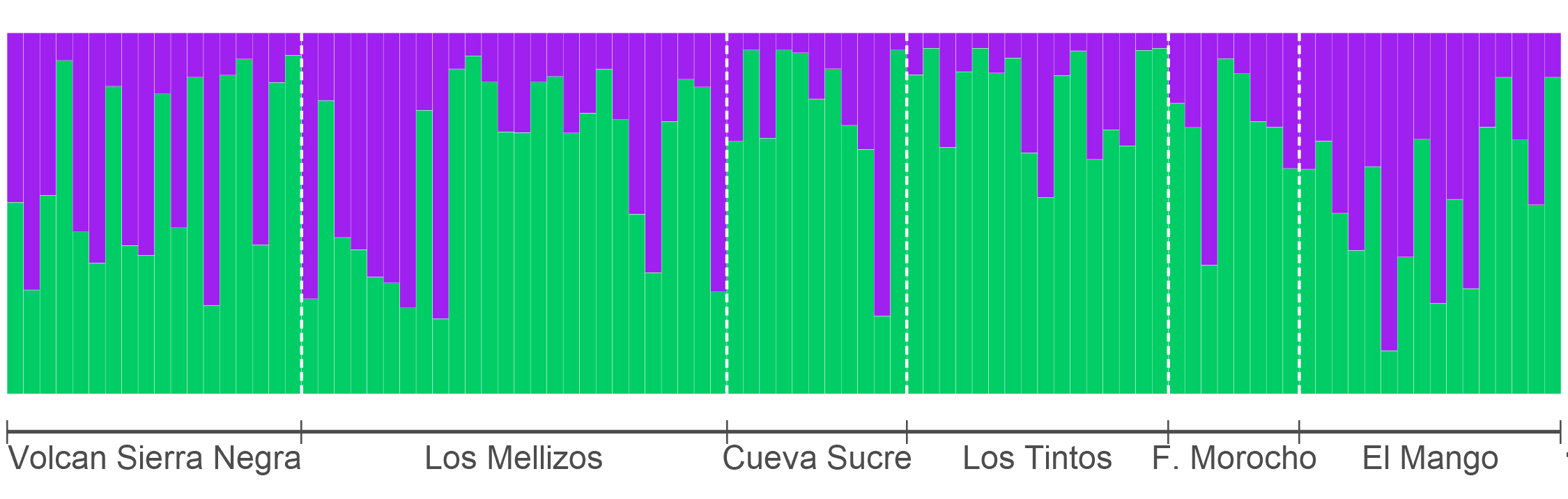

Supplement: S2 Fig — The results are indicated for K = 2, this being the optimum K value (ΔK = 19.62). The values of K correspond to the number of clusters (represented by different colors) in which are grouped the sampled individuals. White dotted lines separate different regions. (TIF) [file pone.0203737.s004.tif]

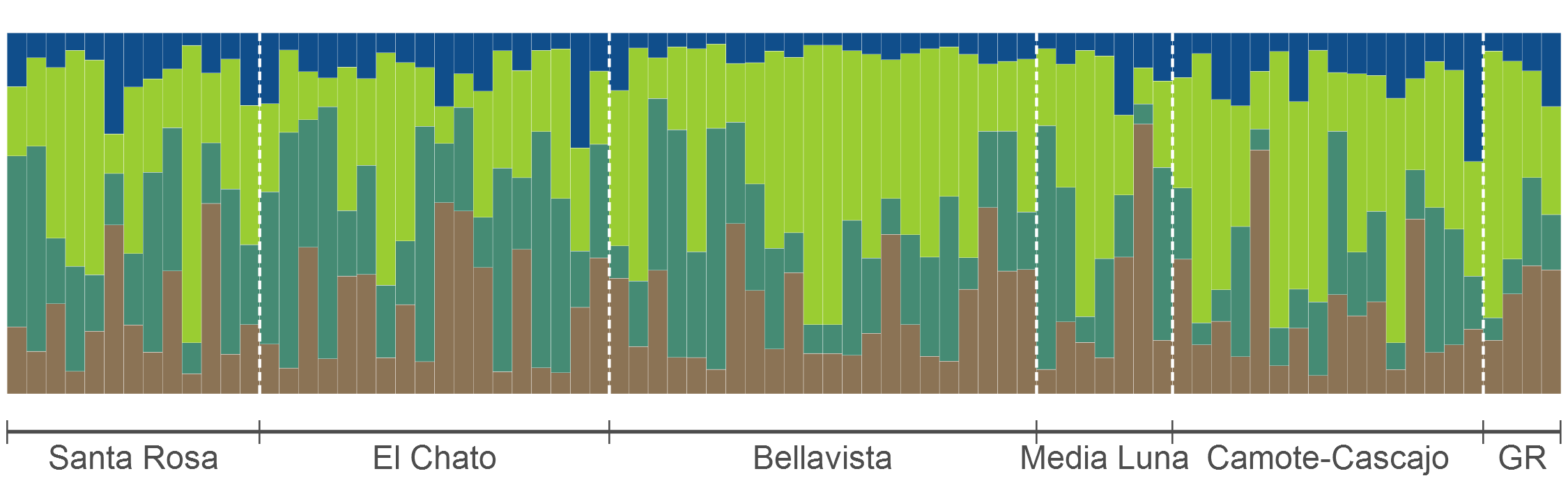

Supplement: S3 Fig — The results are indicated for K = 4, this being the optimum K value (ΔK = 39.57). The values of K correspond to the number of clusters (represented by different colors) in which are grouped the sampled individuals. White dotted lines separate different regions (GR = Granillo Rojo). (TIF) [file pone.0203737.s005.tif]

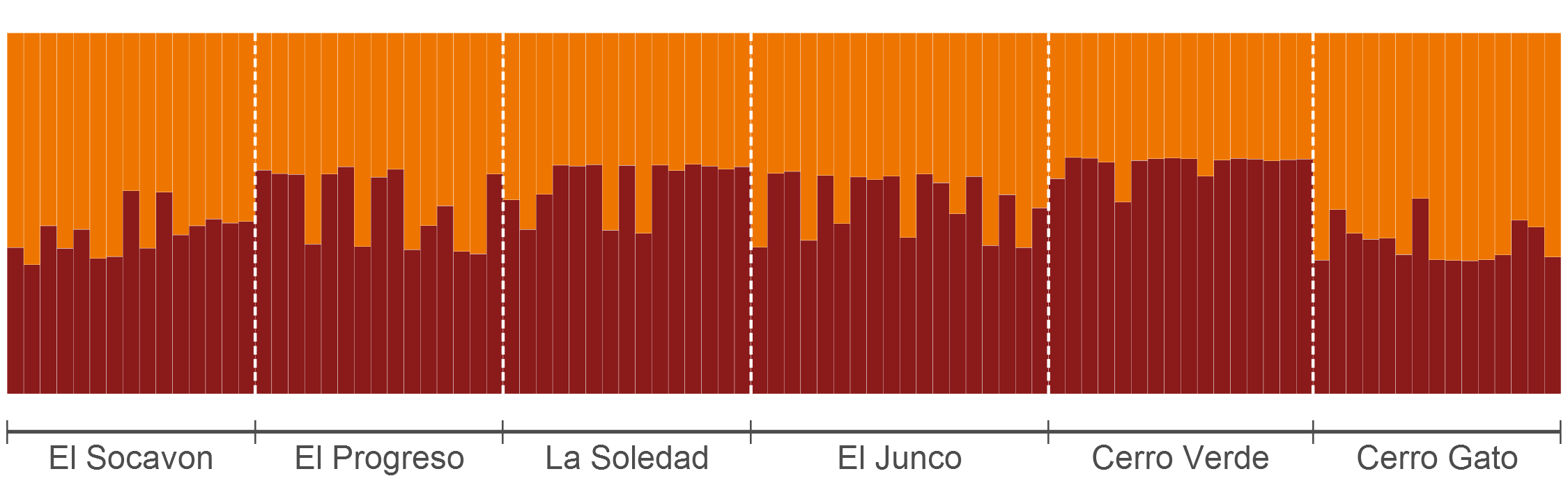

Supplement: S4 Fig — The results are indicated for K = 2, this being the optimum K value (ΔK = 1.83). The values of K correspond to the number of clusters (represented by different colors) in which are grouped the sampled individuals. White dotted lines separate different regions. (TIF) [file pone.0203737.s006.tif]
