# Supplementary material for: Psidium guajava in the Galapagos Islands: Population genetics and history of an invasive species
Source: PLoS One. 2019 Mar 13;14(3):e0203737. doi: 10.1371/journal.pone.0203737 (PMC6415804; doi:10.1371/journal.pone.0203737)
Supplement: S1 Table — (DOCX) [file pone.0203737.s007.docx]

| **Island** | **N** | **% Missing data per primer** | | | | | | | | | | | **%Missing data per island** | **%Total Missing data** |
| --- | --- | --- | --- | --- | --- | --- | --- | --- | --- | --- | --- | --- | --- | --- |
|  |  | **CIR10** | **CIR07** | **CIR05** | **CIR17** | **CIR08** | **CIR11** | **CIR18** | **CIR21** | **CIR09** | **CIR22** | **CIR25** |  |  |
| San Cristobal | 94 | 3.19% | 2.12% | 7.44% | 0% | 0.94% | 0% | 28.72% | 61.70% | 0% | 0% | 0% | 10.30% | 4.53% |
| Santa Cruz | 80 | 0% | 0% | 0% | 0% | 0% | 0% | 0% | 0% | 0% | 0% | 0% | 0% |  |
| Isabela | 95 | 3.15% | 2.10% | 6.31% | 6.31% | 1.05% | 1.05% | 14.73% | 0% | 1.05% | 1.05% | 1.05% | 3.54% |  |
| **Total of individuals** | 269 |  |  |  |  |  |  |  |  |  |  |  |  |  |
