# Supplementary material for: Psidium guajava in the Galapagos Islands: Population genetics and history of an invasive species
Source: PLoS One. 2019 Mar 13;14(3):e0203737. doi: 10.1371/journal.pone.0203737 (PMC6415804; doi:10.1371/journal.pone.0203737)
Supplement: S2 Table — The names of the linked loci appear in rows and columns, whereas population names appear as entries in the table. ISA = Isabela population; SCY = San Cristobal population. No LD was found in Santa Cruz. (DOCX) [file pone.0203737.s008.docx]

|  | mPgCIR10 | mPgCIR07 | mPgCIR05 | mPgCIR17 | mPgCIR08 | mPgCIR11 | mPgCIR18 | mPgCIR21 | mPgCIR09 | mPgCIR22 | mPgCIR25 |
| --- | --- | --- | --- | --- | --- | --- | --- | --- | --- | --- | --- |
| mPgCIR10 | - |  |  |  |  |  |  |  |  |  |  |
| mPgCIR07 |  | - |  |  |  |  |  |  |  |  |  |
| mPgCIR05 |  |  | - |  |  |  |  |  |  |  |  |
| mPgCIR17 |  | ISA | ISA | - |  |  |  |  |  |  |  |
| mPgCIR08 |  |  | ISA |  | - |  |  |  |  |  |  |
| mPgCIR11 |  | ISA |  |  |  | - |  |  |  |  |  |
| mPgCIR18 |  |  |  |  |  |  | - |  |  |  |  |
| mPgCIR21 |  |  |  |  |  |  | ISA, SCY | - |  |  |  |
| mPgCIR09 |  |  | ISA |  | ISA |  | ISA |  | - |  |  |
| mPgCIR22 |  |  |  |  |  | ISA |  |  |  | - |  |
| mPgCIR25 |  |  | ISA |  | ISA |  |  |  | ISA |  | - |
