# Supplementary material for: Psidium guajava in the Galapagos Islands: Population genetics and history of an invasive species
Source: PLoS One. 2019 Mar 13;14(3):e0203737. doi: 10.1371/journal.pone.0203737 (PMC6415804; doi:10.1371/journal.pone.0203737)
Supplement: S3 Table — Results shown correspond to those after Bonferroni correction. (DOCX) [file pone.0203737.s009.docx]

|  | Isabela | Santa Cruz | San Cristobal |
| --- | --- | --- | --- |
| mPgCIR10 | *** | *** | >0.05 |
| mPgCIR07 | *** | *** | >0.05 |
| mPgCIR05 | *** | *** | *** |
| mPgCIR17 | *** | *** | *** |
| mPgCIR08 | *** | * | * |
| mPgCIR11 | *** | *** | *Monomorphic* |
| mPgCIR18 | *** | *** | >0.05 |
| mPgCIR21 | *** | *** | ** |
| mPgCIR09 | *** | *** | >0.05 |
| mPgCIR22 | >0.05 | *Monomorphic* | *Monomorphic* |
| mPgCIR25 | *** | *Monomorphic* | *Monomorphic* |
