# Supplementary material for: Psidium guajava in the Galapagos Islands: Population genetics and history of an invasive species
Source: PLoS One. 2019 Mar 13;14(3):e0203737. doi: 10.1371/journal.pone.0203737 (PMC6415804; doi:10.1371/journal.pone.0203737)
Supplement: S4 Table — The mean frequencies over the three populations are shown as well. (DOCX) [file pone.0203737.s010.docx]

| **Locus** | **Isabela** | **Santa Cruz** | **San Cristobal** | **Mean** |
| --- | --- | --- | --- | --- |
| **mPgCIR10** | 0.127 | 0.131 | 0.041 | 0.100 |
| **mPgCIR07** | 0.233 | 0.188 | 0.082 | 0.168 |
| **mPgCIR05** | 0.147 | 0.150 | 0.198 | 0.165 |
| **mPgCIR17** | 0.239 | 0.179 | 0.178 | 0.199 |
| **mPgCIR08** | 0.138 | 0.113 | 0.069 | 0.107 |
| **mPgCIR11** | 0.255 | 0.218 | 0.001 | 0.158 |
| **mPgCIR18** | 0.160 | 0.179 | 0.098 | 0.146 |
| **mPgCIR21** | 0.153 | 0.209 | 0.193 | 0.185 |
| **mPgCIR09** | 0.159 | 0.202 | 0.035 | 0.132 |
| **mPgCIR22** | 0.028 | 0.001 | 0.001 | 0.010 |
| **mPgCIR25** | 0.190 | 0.000 | 0.001 | 0.064 |
