# Supplementary material for: Psidium guajava in the Galapagos Islands: Population genetics and history of an invasive species
Source: PLoS One. 2019 Mar 13;14(3):e0203737. doi: 10.1371/journal.pone.0203737 (PMC6415804; doi:10.1371/journal.pone.0203737)
Supplement: S5 Table — Missing data was ignored for this analysis. (DOCX) [file pone.0203737.s011.docx]

|  | **Isabela** | | **Santa Cruz** | | **San Cristóbal** | |
| --- | --- | --- | --- | --- | --- | --- |
| **Source of variation** | **% of variation** | ***p*-value** | **% of variation** | ***p*-value** | **% of variation** | ***p*-value** |
| Between regions within the island | 3.56 | 0.005 | 0.80 | 0.242 | 2.97 | 0.002 |
| Between samples within regions | 60.19 | 0.001 | 53.29 | 0.001 | 30.63 | 0.001 |
| Within samples | 36.25 | 0.001 | 45.91 | 0.001 | 66.40 | 0.001 |
