# Supplementary material for: Psidium guajava in the Galapagos Islands: Population genetics and history of an invasive species
Source: PLoS One. 2019 Mar 13;14(3):e0203737. doi: 10.1371/journal.pone.0203737 (PMC6415804; doi:10.1371/journal.pone.0203737)
Supplement: S6 Table — (DOCX) [file pone.0203737.s012.docx]

|  | **Volcan Sierra Negra** | **El Cura** | **Los Mellizos** | **Los Ceibos** | **Los Tintos** | **Barrio Loja** |
| --- | --- | --- | --- | --- | --- | --- |
| **Volcan Sierra N.** | - |  |  |  |  |  |
| **El Cura** | -0.004 | - |  |  |  |  |
| **Los Mellizos** | -0.003 | 0.053 | - |  |  |  |
| **Los Ceibos** | 0.009 | 0.065 | -0.008 | - |  |  |
| **Los Tintos** | 0.036 | 0.085 | 0.066 | 0.034 | - |  |
| **Barrio Loja** | 0.006 | 0.084 | -0.036 | -0.009 | 0.019 | - |
| **El Mango** | 0.012 | -0.006 | 0.088 | 0.053 | 0.116 | 0.098 |
