# Supplementary material for: Psidium guajava in the Galapagos Islands: Population genetics and history of an invasive species
Source: PLoS One. 2019 Mar 13;14(3):e0203737. doi: 10.1371/journal.pone.0203737 (PMC6415804; doi:10.1371/journal.pone.0203737)
Supplement: S7 Table — (DOCX) [file pone.0203737.s013.docx]

|  | **Santa Rosa** | **El Chato** | **Bellavista** | **Media Luna** | **Camote** | **Cascajo** |
| --- | --- | --- | --- | --- | --- | --- |
| **Santa Rosa** | - |  |  |  |  |  |
| **El Chato** | -0.007 | - |  |  |  |  |
| **Media Luna** | 0.004 | -0.023 | - |  |  |  |
| **Bellavista** | -0.016 | 0.004 | -0.006 | - |  |  |
| **Cascajo** | -0.005 | 0.060 | 0.022 | -0.019 | - |  |
| **Camote** | 0.022 | 0.027 | -0.055 | -0.006 | 0.035 | - |
| **Granillo Rojo** | 0.083 | 0.060 | -0.022 | 0.080 | 0.089 | 0.008 |
