# Supplementary material for: Psidium guajava in the Galapagos Islands: Population genetics and history of an invasive species
Source: PLoS One. 2019 Mar 13;14(3):e0203737. doi: 10.1371/journal.pone.0203737 (PMC6415804; doi:10.1371/journal.pone.0203737)
Supplement: S8 Table — (DOCX) [file pone.0203737.s014.docx]

|  | **El Socavon** | **El Progreso** | **La Soledad** | **El Junco** | **Cerro Verde** |
| --- | --- | --- | --- | --- | --- |
| **El Socavon** | - |  |  |  |  |
| **El Progreso** | -0.014 | - |  |  |  |
| **La Soledad** | 0.075 | 0.026 | - |  |  |
| **El Junco** | 0.039 | 0.031 | -0.009 | - |  |
| **Cerro Verde** | 0.071 | 0.029 | 0.022 | 0.041 | - |
| **Cerro Gato** | -0.045 | -0.001 | 0.076 | 0.037 | 0.049 |
